# Progress, Advances and Challenges in Bipolar Disorder

**DOI:** 10.31083/AP49937

**Published:** 2026-06-17

**Authors:** Gin S. Malhi, Kinga Szymaniak, Gurubhaskar Shivakumar, Erica Bell

**Affiliations:** ^1^Academic Department of Psychiatry, Kolling Institute, Northern Clinical School, Faculty of Medicine and Health, The University of Sydney, Sydney, NSW 2065, Australia; ^2^CADE Clinic and Mood-T, Royal North Shore Hospital, Northern Sydney Local Health District, St. Leonards, NSW 2065, Australia; ^3^Department of Psychiatry, University of Oxford, OX3 7JX Oxford, UK; ^4^Uehiro Oxford Institute, Faculty of Philosophy, University of Oxford, OX1 1PT Oxford, UK; ^5^Adult Mental Health Unit, Hornsby Ku-Ring-Gai Hospital, Northern Sydney Local Health District, Hornsby, NSW 2077, Australia

## 1. Introduction

In psychiatry, the vast majority of diagnoses are descriptive and rely solely on clinical phenomenology, and bipolar disorder is no exception. Having appeared only recently in the diagnostic nomenclature in comparison to its near relation (melancholia), bipolar disorder began its journey as ‘manic-depressive insanity’—a description that captured the two extremes of the illness [1]. However, in addition to its cross-sectional characteristics, Kraepelin also attached importance to the course of an illness and used this to distinguish between different types of illness.

He characterised manic-depressive insanity as an episodic illness punctuated by periods of recovery and observed that *dementia praecox*, the forerunner of schizophrenia, was more prone to persist as a psychosis that led to gradual deterioration over time [2]. Consequently, these broad groupings of dementia praecox and manic-depressive insanity, introduced in 1899 by Kraepelin and often referred to as the ‘Kraepelinian Dichotomy’, include many psychiatric conditions that have since been defined as members of the psychotic and mood disorders domains, respectively [3, 4]. Within the mood disorders domain recurrent depressive episodes are classified as ‘major depressive disorder’, of which melancholia is a subtype. However, the key subdivision of mood disorders is the separation of ‘manic-depressive illness’, also recurrent in nature, from major depression, a distinction that is based on the presence of manic symptoms occurring as episodes. Subsequently, the term ‘bipolar disorder’ has replaced manic-depressive illness, which was used initially by Karl Leonhard in 1957, and was formally introduced in Diagnostic and Statistical Manual of Mental Disorders, 3rd Edition (DSM-III) in 1980 to emphasise its phases referred to colloquially as ‘highs’ and ‘lows’ [5].

Since these initial subdivisions, further phenomenological separation of major depression and bipolar disorder has not been possible. While mania does seem to distinguish those who have recurrent mania in addition to depression, the lows of bipolar disorder are clinically indistinguishable from those of major depression. In trying to obtain a more granular clinical picture of depression, researchers have examined both ‘major depression’ and ‘bipolar depression’ (i.e., that which occurs in the context of bipolar disorder), and many have suggested that there are distinguishing characteristics that define both ‘depressions’. However, none have withstood scrutiny, and neither research nor clinical experience has been able to identify reliable features that differentiate the two entities.

One might be wondering why we raise this long-standing and unresolved problem in the current themed issue that wishes to champion the progress and advances in bipolar disorder. The reason is that while there has been significant progress in establishing a clearer picture of this psychiatric phenomenon, and this indeed should be celebrated, the separation of bipolar disorder from major depressive disorder is a fundamental challenge that is likely to hinder a deeper understanding of mood disorders. Therefore, we feel that unless we solve this conundrum, we will not be able to make meaningful progress.

## 2. A Longitudinal Perspective

**Fig. 1.**
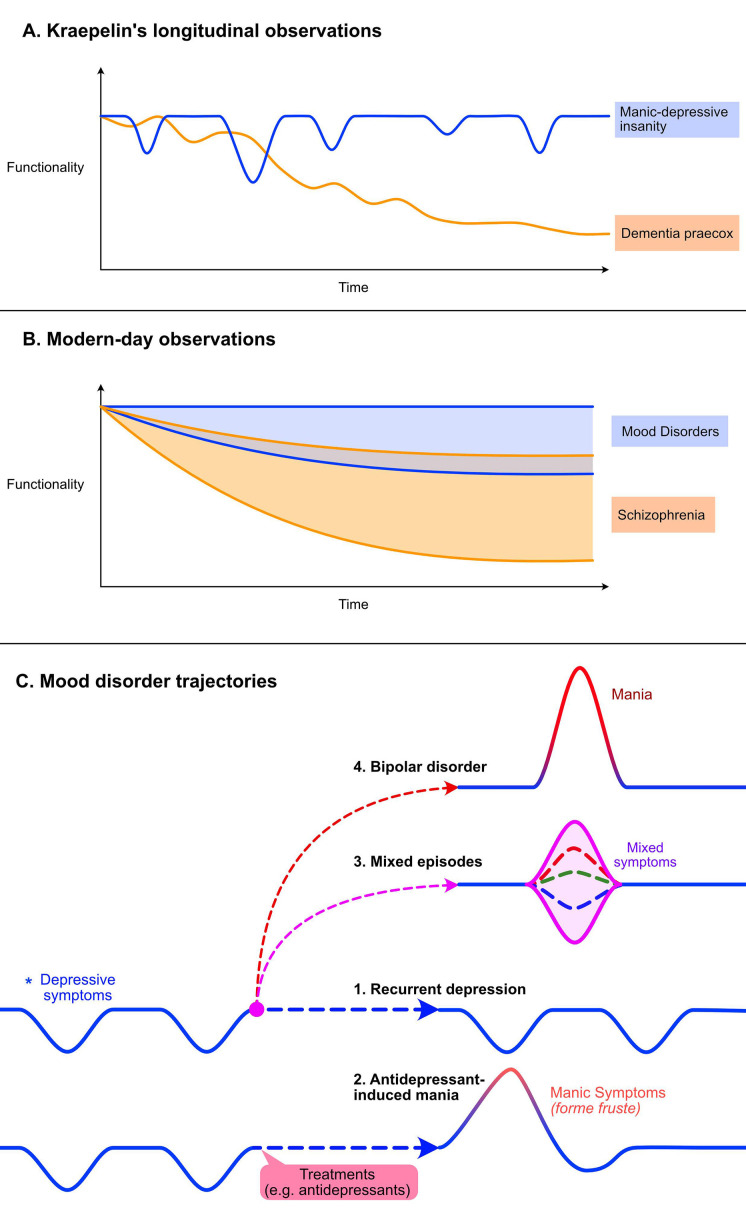
**Longitudinal perspectives on mood disorders**. (A) An adaptation of Kraepelin’s longitudinal observations of his patients that, when viewed cross-sectionally, experienced both affective and psychotic symptoms. When viewed longitudinally, two general groupings of patients were apparent—those who experienced a deteriorating trajectory in terms of their functionality, and those whose functionality remained relatively consistent over time, albeit with episodes of severe illness. Kraepelin termed these two groups ‘dementia praecox’ and ‘manic-depressive insanity’ respectively, and these encompass what we now term as schizophrenia and mood disorders, respectively. (B) The change in functioning in mood disorders and schizophrenia is shown, reflecting the findings of recent research, which has revealed, contrary to expectations, that some patients with mood disorders have significant and sustained functional impairment over the course of their life, while a proportion of those with schizophrenia manage to recover and retain reasonable functioning [7, 8]. Thus, both patient groups generally experience a broader range of potential functional impairment that is overlapping and less distinct than that observed by Kraepelin. (C) Shows our current understanding of the illness trajectories of those with mood disorders. Initially, the majority of patients present with episodes of depression, then the individual may continue to (1) experience recurrent depressive episodes (recurrent depression), which may be treated with antidepressants. (2) However, for a subset of patients, the administration of an antidepressant can precipitate manic symptoms, which may in fact be a *forme fruste* of an incipient bipolar disorder. (3) Other patients may instead go on to experience recurrent symptoms from across the full mood spectrum, i.e., mixed episodes of mood (purple shading). Previously, these symptoms have been conceptualised as belonging to three domains: activity, cognition, and emotion (ACE) [9], and these three domains are shown as green, blue, and red dashed lines, respectively, within the mixed mood episode. (4) Finally, a proportion of individuals will go on to experience acute manic symptoms that constitute an episode of mania, and thus a diagnosis of bipolar disorder is established.

A key advance in Kraepelin’s conceptualisation of psychiatric phenomenology was the longitudinal approach to examining his patients. This allowed him to observe patterns of symptoms and illness progression that differentiated patients based on the course and outcomes of their illness. In those with dementia praecox (now termed ‘schizophrenia’), Kraepelin noted a deteriorating course of the illness, wherein a patient’s functionality and severity of symptoms worsened over time when observed over a number of years. In contrast, in those who he classified as having affective psychoses (including those with what we now term ‘bipolar disorder’), although there were acute exacerbations of their illness in the form of mood episodes, overall, there was no long-term deterioration in functioning (see Fig. 1A) [6]. The divergent trajectories of these two groups of patients over time led to the delineation of those with a primarily psychotic illness (i.e., schizophrenia) from those with a predominantly affective illness (i.e., mood disorders) (see Fig. 1B, Ref. [7, 8]), even though many of Kraepelin’s patients experienced symptoms from both domains (i.e., both psychotic and mood symptoms). This redefinition of illnesses warranted the longitudinal examination of patients and tracking of psychiatric phenomenology in a chronological manner. This allowed for a more nuanced understanding of how these two groups of illnesses develop and progress and how they differ from each other—a distinction that would not have been made if patients were only assessed cross-sectionally. Therefore, the course of an illness over time is a critical factor when examining psychiatric diagnoses, and in particular mood disorders, which we now understand are mostly recurrent and chronic illnesses.

Within the group of mood disorders, we also see differences in trajectories of different illnesses in patients when they are followed longitudinally. Several key advances in our understanding of bipolar disorder have been made using longitudinal and follow-up studies. One key advance that has stemmed from developments in the treatment of mood disorders is the understanding that for a subset of patients with depression, the administration of antidepressants can lead to manic symptoms. In addition, mixed states are now recognized as a separate ‘kind’ of mood episode, that is different from agitated depression. Thus, when following patients with depression, several trajectories emerge (see Fig. 1C, Ref. [9]): there are those who maintain a lifelong trajectory of only depressive symptoms and episodes without experiencing any manic symptoms (i.e., recurrent major depressive disorder). This is the majority of patients, and they differ from those who have bipolar disorders (manic symptoms/episodes); those who go on to experience manic episodes (bipolar disorder), which can occur either spontaneously as part of bipolar disorder or because of antidepressant treatments; and those who experience mixed symptoms where mania and depression overlap. Despite our understanding of these differences, we still lack the ability to reliably separate—prior to the manifestation of manic symptoms—those who will go on to develop mania in the future (and therefore have what we currently consider to be bipolar disorder) from those who will not. In other words, when these patients are experiencing depressive episodes early in the course of their illness (see * in Fig. 1C), we have no way of identifying if their depressive symptoms will be part of a future bipolar disorder that is yet to unfold and what the trajectory of their illness will look like. At this early stage of the illness, the clinical phenomenology is indistinguishable, and thus, we remain unable to intervene and modify the course of a patient’s illness until a breakthrough manic episode occurs.

## 3. Searching for a Signal

Over the past forty years, advances in our ability to interrogate psychiatric disorders, using genetics, imaging techniques, and neuropsychological assessments, have meant that some signals to distinguish mood disorders and phases of illness have been uncovered. For example, heritability and genetic signals that point to bipolar disorder [10], structural and functional changes that occur in different phases, and cognitive impacts of the illness have all been determined from recent research [11]. However, these investigations are mostly based on the assumption that patients with unipolar depression and those with bipolar disorder are two different groups. This is problematic as it fails to reveal any potential signal that could help us uncover a clear biomarker separating these two groups of patients, allowing us to move away from relying solely on clinical phenomenology. Therefore, we are still highly dependent on patients’ subjective experiences and clinical observations to detect and diagnose mood disorders. Because of this, patients who present initially with depression do not receive a diagnosis of bipolar disorder until a manic episode occurs. This delay means that effective treatments that may have prevented acute episodes of mania may be unnecessarily withheld until manic symptoms manifest. This can lead to functional impairment and putative neurobiological and psychological insults from the illness itself that may be potentially irreversible.

At the same time, the treatment of bipolar depressed patients with antidepressants prior to making a definitive diagnosis of bipolar disorder (contingent on mania) has meant that many patients do not receive effective treatment and therefore do not respond. These non-responders have been described as having depression that is ‘difficult to treat’, ‘treatment-refractory’, or ‘treatment-resistant’ [12]. The latter, treatment resistant depression (TRD), is presently the most widely used term. It captures a significant proportion of patients, largely because of the low threshold of non-response needed to acquire this label (two failed antidepressant trials), and consequently, the TRD ‘population’ is highly heterogeneous [13]. Nevertheless, the label serves as a useful means to group patients who are non-responders and whose condition can perhaps be regarded as a bridge between major depressive disorder and bipolar disorder. This group of non-responders to antidepressants also provides another opportunity to study patients with depression, for example, investigating their course of illness to determine if and when they transfer to bipolar disorder. These patients will include those who have treatment-induced manic symptoms. Differentiating between these two subgroups is also critically important and further underscores the need to examine TRD. TRD features that could potentially be examined along with its clinical phenomenology include the pattern of symptoms longitudinally, as well as neurobiological characteristics and treatment responsiveness across the full spectrum of mood disorder interventions.

This stagnation in progress due to our dependence on clinical phenomenology for diagnosis means that a new approach is urgently needed, and one where long-held assumptions are systematically challenged and examined, specifically, the assumption that bipolar disorder and major depression are two different illnesses. By adopting an open perspective, this new approach should acknowledge the fact that bipolar disorder and major depression are clearly strongly linked, at least phenomenologically, but also that this may hint at the possibility that these disorders are, in fact, intertwined at a deeper level, such as within neurobiological structures. Kraepelin integrated multiple mood states within his description of manic-depressive insanity, and perhaps this description may retain its value in linking depression and bipolar disorder. It may be that these illnesses have the same underlying pathology or insult that, due to factors we do not yet fully understand, results in a divergence of trajectories and the inception of mania. For example, both illnesses may involve the same pathobiological insult to a specific brain structure or network, but because of the severity of this insult, its exact location, or other moderating factors such as genetic vulnerabilities or exposures to specific environmental triggers, some individuals may go on to develop mania, whereas others will not. For now, we do not know whether bipolar depression or unipolar depression is merely a phenocopy of the other or has the same etiology. Therefore, these questions must be first answered if we are to substantially advance the detection and treatment of bipolar disorder.

## 4. A Way Forward

In order to systematically investigate the overlap between bipolar disorder and unipolar depression, it is critical that three key steps be undertaken. First, the population of patients examined must have clear-cut major depression or bipolar disorder. In particular, there should be no suspicion that their illness is caused by immediate external precipitants such as the administration of an antidepressant (i.e., antidepressant-induced mania), and that the mood episodes experienced have only entailed symptoms from one ‘pole’ of illness (i.e., they have not experienced mixed states or agitated depression). By focusing our efforts on these two patient groups, which appear to have separated from each other with regard to the presence of manic symptoms and conceptually have the least overlap when compared to those with mixed symptoms or antidepressant-induced mania, the likelihood of finding other differences between bipolar disorder and unipolar depression is maximised.

Second, patients who do experience clear-cut mania (that is, exclusively manic symptoms without a mixed presentation) should then be followed longitudinally, and their future depressive episodes should be closely examined and compared to those with unipolar depression to uncover any phenomenological differences. This will help us determine whether our current clinical tools and understanding of these two illnesses are accurate and precise enough to detect a difference if there is one. Finally, following this examination, if a signal is found, retrospective re-evaluation of past databases can occur. This refers to the interrogation of datasets wherein patients were recruited when they had only a diagnosis of depression (i.e., early in their illness course). This is potentially useful because we now understand that a proportion of these patients may go on to experience manic symptoms and thus later in life be diagnosed with bipolar disorder. Therefore, having been assessed and diagnosed with depression alone, they may serve as harbingers of signals that indicate the development of bipolar depression and serve as forerunners of this illness. However, this research will not be easy, and it may still not yield sufficient insight. Nevertheless, the current trajectory of this research has arguably reached its zenith, and it is perhaps time for a more ambitious agenda.

## 5. Conclusions

Our understanding of bipolar disorder has advanced over the years, and we now know much more about this disorder than ever before. However, its ‘muddied’ relationship with major depressive disorder likely prevents us from advancing further. We still don’t know to what extent these two disorders are related—are they cousins or ‘identical twins’? Currently, cross-sectional assessments and episodic treatments are based on symptom profiles from the milieu of clinical and research practices in mood disorders. As a result, treatments for patients with mood disorders continue to focus on short-term symptomatic relief, with little clear evidence that they meaningfully modify the course of illness or improve the prognosis for patients. However, if a broader perspective is adopted, wherein long-held assumptions are closely examined, and a new, systematic research of the relationship between mood disorders is undertaken, then perhaps significant advances in our understanding of these chronic and devastating illnesses can be made.
